# Time-resolved magnetic resonance angiography (TR-MRA) for the evaluation of post coiling aneurysms; A quantitative analysis of the residual aneurysm using full-width at half-maximum (FWHM) value

**DOI:** 10.1371/journal.pone.0203615

**Published:** 2018-09-07

**Authors:** Ayako Ikemura, Ichiro Yuki, Hiroaki Suzuki, Tomoaki Suzuki, Toshihiro Ishibashi, Yukiko Abe, Mitsuyoshi Urashima, Chihebeddine Dahmani, Yuichi Murayama

**Affiliations:** 1 Department of Neurosurgery, The Jikei University Hospital, Tokyo, Japan; 2 Department of Radiology, The Jikei University Hospital, Tokyo, Japan; 3 Department of Molecular Epidemiology, The Jikei University School of Medicine, Tokyo, Japan; 4 Siemens Healthineers Japan, Tokyo, Japan; Universitatsklinikum Freiburg, GERMANY

## Abstract

Magnetic resonance image (MRI) is now widely used for imaging follow-up for post coiling brain aneurysms. However, the accuracy on the estimation of residual aneurysm, which is crucial for the retreatment planning, remains to be controversial. The purpose of this study is to evaluate a new post-processing technique that provides improved estimation of the residual aneurysm after coil embolization. One hundred aneurysms on 93 patients who underwent coil embolization for brain aneurysm were evaluated using the 1.5 Tesla time-resolved magnetic resonance angiography (TR-MRA) one year after the treatment. To minimize the inter-observer variability caused by the window level adjustment, an automatic post processing protocol using the full-width at half-maximum (FWHM) value was utilized. The result was then compared with that from the conventional cerebral angiography. Of the 97 aneurysms that underwent both TR-MRA and DSA, 23 (23.7%) showed residual neck / dome during follow-up. After window level adjustment, the size of the parent artery in the TR-MRA was consistent with that in the DSA. The reconstructed Volume Rendering images provided clear contours of the residual aneurysms and contributed to the understanding the configuration of residual aneurysm. The largest and the smallest diameter of the residual aneurysms was larger in the TR-MRA than in the DSA (8.05 vs. 7.72 mm, *p* = 0.0004; 4.99 vs. 4.19 mm, *p* = 0.007 respectively). The sensitivity, specificity, and positive and negative predictive values of TR-MRA compared to DSA were 100%, 97%, 73%, and 100%, respectively. Using the FWHM value to optimize the window level adjustment, the size of the residual component observed in the TR-MRA was larger compared to that in the DSA whereas the size of neck and the parent artery showed consistency between the two modalities. This image processing technique can be used as an effective screening tool for evaluating residual component in post-coiling brain aneurysms.

## Introduction

Precise measurement on the “shape and size” of the residual component in a previously treated aneurysm is crucial in deciding for the re-treatment plan. Studies have indicated that time-resolved 3D contrast-enhanced magnetic resonance angiography (TR-MRA) is suitable for evaluating aneurysms previously treated with coil embolization or stenting [1, 2] because it is relatively insusceptible to metal artifacts compared to other sequences, such as time of flight (TOF) MRA. It also reduces the influence of venous contamination, which is one of the limitations of conventional contrast-enhanced MRA (CE-MRA). However, little is known about how the TR-MRA images of residual aneurysm compare to those of conventional digital subtraction angiogram (DSA). In addition, little has been reported on the optimal post-processing method to delineate residual aneurysms. In fact, the variability that occurs during the process of window level adjustment can lead to inconsistent measurement values, thus causing a potential risk of under / over estimation of aneurysm recanalization.

The full-width at half-maximum (FWHM) value, which is a commonly used measure in the field of electrical engineering, has been used for the quantitative measurements of images on computed tomography (CT) angiography or contrast-enhanced magnetic resonance imaging (MRI) to increase the accuracy of the quantitative data [3–5]. The purpose of this study is to evaluate a new post-processing technique for the TR-MRA that provides improved accuracy on the estimation of residual aneurysm by utilizing a program with FWHM algorithm.

For qualitative analysis, detection sensitivity of the TR-MRA compared to the conventional DSA was evaluated. For quantitative analysis, measurement values of each residual aneurysm, as well as the size of parent artery, were compared between the two imaging modalities and statistical analysis was performed.

## Materials and methods

### Coil embolization of brain aneurysms

Between July 2012 and March 2015, a total of 379 aneurysms were treated with coil embolization at the Jikei University Hospital. Of 287 patients with follow-up angiography after one year, 93 (100 aneurysms) who also underwent TR-MRA were included. The hospital’s institutional review board (IRB) approved the study.

Endovascular coil embolization of each aneurysm was done using the angiographic system, AXIOM Artis dBA^®^ (Siemens Healthcare GmbH, Erlangen, Germany). For those with wide neck aneurysms, the double catheter technique or balloon-assisted technique was primarily used, while the stent-assisted technique was selected as a rescue plan in case other techniques failed to prevent coil herniation into the parent artery. This work was approved by the ethics committee of the Jikei University School of Medicine. The approval number is clinical research 27–236 (8121). Both verbal and written consent was obtained for all patients before the treatment.

### Pre- and post-procedural DSA for evaluating residual aneurysm

On the day of treatment, conventional 2D-DSA and 3D-DSA acquisitions were performed once the guiding catheter was positioned at the cervical segment of the internal carotid artery (or vertebral artery). For the acquisition protocol of the 3D-DSA, the motorized frontal C-arm, typically used for 3D rotational angiography, was used to acquire 496 projection images over a 200° arc (rotation time, 5 sec) at 80 kV (peak). Moreover, 18 cc of diluted contrast material (60%) was injected at an injection rate of 3.0 cc/sec over 5 sec. The injection delay time was set to 1.5 seconds and the 3D-DSA was repeated at the end of treatment.

The acquired images were further processed by an image-processing application, Aquarius iNtuition^®^, Version 4.4.7.108.0 (TeraRecon, Foster City, USA). Follow-up DSA was repeated 12 months after the treatment as a routine procedure, using the same acquisition protocol.

### Post-treatment imaging follow-up using MRI

Routine post-treatment MRI, including TOF MRA, was performed one day after using the 1.5T MR imaging system (Symphony^®^; Siemens Healthcare GmbH, Erlangen, Germany), with a 4-channel head coil. This was repeated at 3 and 6 months post-treatment. Subsequently, TR-MRA was performed at approximately one year after the treatment. The imaging parameters used for the acquisition were as follows: TR/TE, 2.36 msec / 0.94 msec; flip angle, 30°; FOV, 220 mm; matrix, 224 x 175; slice thickness, 1.0 mm; bandwidth, 970 KHz; and the parallel imaging method with k-space sensitivity encoding “GRAPPA” (Siemens Healthcare GmbH, Erlangen, Germany), 2. A coronal or a sagittal 20-mm slab was acquired, depending on the relation between the parent artery and the aneurysm.

Ten dynamic scans were obtained at a temporal resolution of 1.23 sec, with voxel size of 1.0 x 1.0 x 1.0 mm and total scan time of 40 sec. A monophasic injection protocol was implemented, with automatic power injection (Sonic shot GX^®^; Nemoto Kyorindo, Tokyo, Japan) of contrast medium: 0.2 mL x body weight of Gadodiamide Hydrate (Omniscan^®^; Daiichi Sankyo Healthcare, Tokyo, Japan). This was injected at a flow rate of 2 mL/sec, followed by a saline flush of 20 mL. The TR-MRA was initiated 8 sec after the start of injection of the contrast medium.

### Creation of 3D images of TR-MRA using the FWHM value

The image data obtained from TR-MRA was reconstructed using a 3D workstation, Aquarius iNtuition^®^. To minimize inter-observer variability that occurs during the window level adjustment, the FWHM value was used to measure the size of each aneurysm/residual aneurysm [3–5]. First, the optimal phase of contrast filling in the target vessel was selected based on the phase sampling view. The extracted DICOM data was then transferred to the 3D workstation and the initial reconstructed images of the treated aneurysm was created using the multi-planar reconstruction (MPR) images.

**Fig 1:**
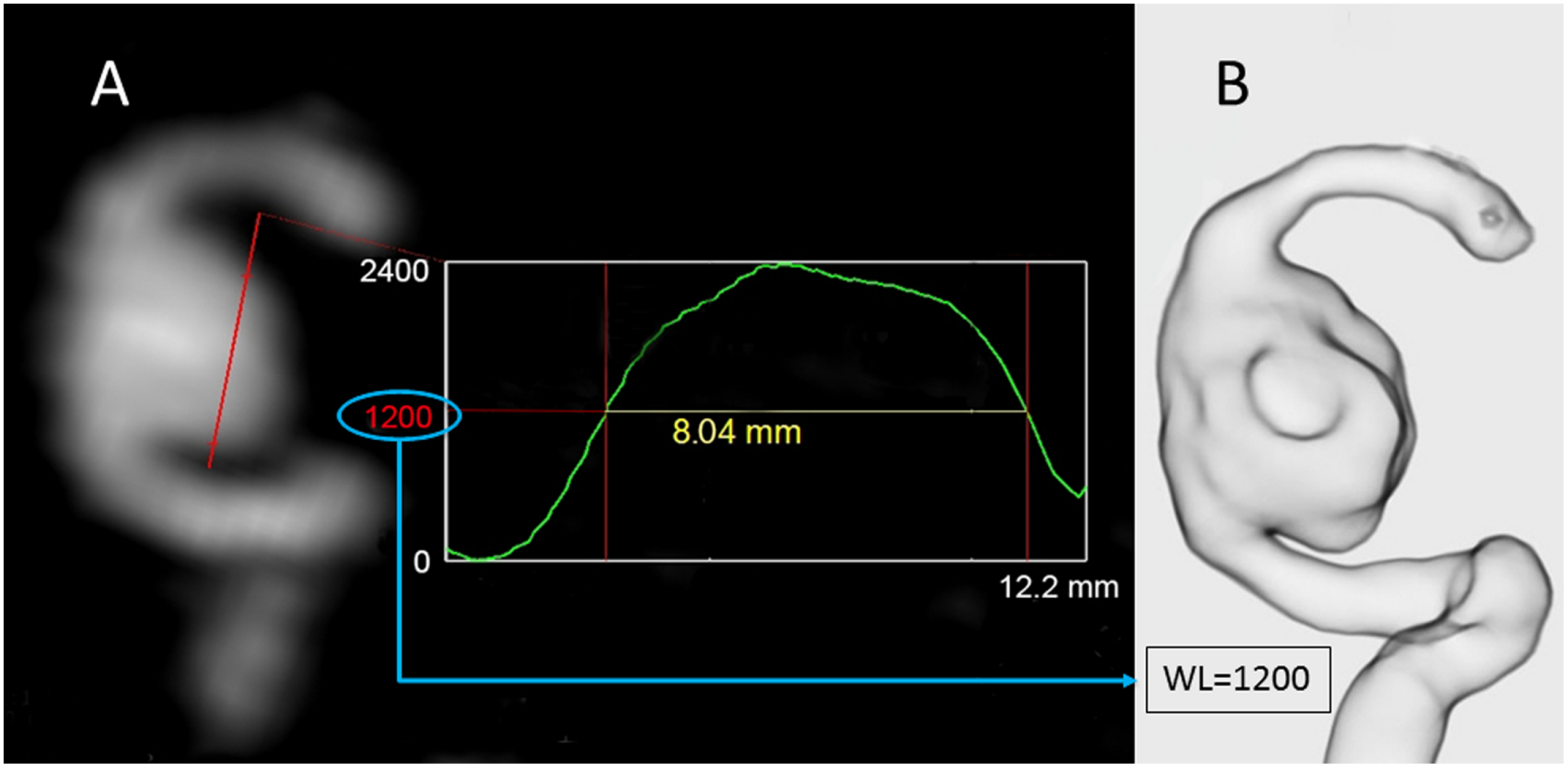
Window level adjustment based on the full-width at half-maximum value (FWHM). **(A)** Using an MPR image, a signal-intensity-profile along a line crossing the residual aneurysm was obtained. The FWHM value was calculated based on the signal-intensity-profile. The calculated value was then assigned to adjust the “window level (WL)” for the Volume Rendering (VR) reconstruction. **(B)** The reconstructed VR image (translucent view) showed the clear contours of the residual aneurysm.

The MPR image with the optimal projection was then selected and a signal intensity profile along a line crossing the residual aneurysm was obtained (Fig 1). Lastly, an FWHM value was calculated based on the signal intensity profile and the value was assigned to the “window level” (WL) of the VR reconstruction parameters. The pre-set VR pattern of “translucent view” was selected to minimize variabilities caused by the different VR setting parameters.

### Qualitative analysis; Detection sensitivity of the TR-MRA compared to DSA

Two neuro-interventionalists (AI and IY) performed the qualitative analysis to evaluate whether the image findings in the reconstructed TR-MRA images, which was performed 1 year after the treatment, were consistent with the 1 year follow-up DSA findings. The evaluators were independent from the decision making of the treatment planning of each patient, thus the evaluations were performed in the blind fashion.

A dataset of anonymized and randomly numbered TR-MRA images was created for the evaluation. Another dataset for DSA images was also created in the same manner. Two neuro-intervetionalits reviewed each dataset individually and assessed using the following classification. The “Major re-canalization” was defined as “Raymond-Roy Occlusion Classification 3 and the observer recommended re-treatment based on the image findings” [6]. Each aneurysm evaluated by TR-MRA was classified into one of four categories in relation to the DSA findings: 1) true positive, or the aneurysm showing major re-canalization on both TR-MRA and DSA; 2) false positive, or the aneurysm showing major re-canalization based on TR-MRA but not on DSA; 3) false negative, or the aneurysm showing major re-canalization based on DSA but not on TR-MRA; and 4) true negative, or the aneurysm not showing major re-canalization on either TR-MRA or DSA.

The sensitivity, specificity, positive predictive value, and negative predictive value, as well as the 95% confidence interval (95%CI) were calculated. The inter-modality agreement was evaluated using kappa statistics to investigate the inter-rater reliability between the two evaluators.

### Quantitative analysis comparing the size of residual aneurysms between the TR-MRA and DSA images

Patients who were found to have a residual neck/aneurysm on one-year follow-up DSA or MRA were selected for the analysis. The size of the residual aneurysm was calculated as follows. First, a 2D DSA image with optimal projection for intra-operative view was selected and a line connecting the most proximal and most distal points of the aneurysm neck was drawn (neck line). The largest and smallest diameters of the residual aneurysm in the same projection was then measured and recorded. The same parameters of the reconstructed TR-MRA image using the same projection were evaluated in the same manner. Lastly, to confirm inter-modality consistency, the size of the parent artery at the proximal neck was compared between the TR-MRA and the 2D DSA.

The measurement values of each residual aneurysm as well as the parent artery were compared between the TR-MRA and 3D-DSA images. The between-group correlation was analyzed using Spearman’s rank correlation coefficient (Spearman’s Rho). To examine differences in size measurements between the two groups, statistical analysis using Wilcoxon signed-rank test was also performed.

## Results

### Patient demographics

One hundred aneurysms in 93 patients (male-to-female ratio 29:64; age, 15–80 years; mean age, 59 years) successfully underwent coil embolization. The location of the aneurysms were the anterior communicating artery (n = 15), anterior cerebral artery (n = 2), middle cerebral artery (n = 15), cavernous internal carotid artery (ICA) (n = 3), para-clinoid ICA (n = 10), ophthalmic artery (n = 11), ICA superior hypophyseal artery (n = 16), posterior communicating artery (n = 12), anterior choroidal artery (n = 4), intracranial vertebral artery (n = 1), basilar trunk (n = 4), and basilar top (n = 7). Both the TR-MRA and DSA were performed approximately one year after the treatment in all of the patients.

Of the 100 aneurysms, 97 were included in the study after three were excluded due to sub-optimal contrast filling in the TR-MRA. The average maximal diameter of the aneurysms was 6.9±3.2 mm before the treatment. Twenty-one aneurysms (21%) were treated by stent-assisted coil embolization, while the rest were treated by the simple technique, double-catheter technique, or balloon-assisted technique. The mean follow-up period after coil embolization was 16.3 and 15.9 months for the DSA and TR-MRA, respectively.

### Reconstructed 3D images of TR-MRA using the FWHM value

**Fig 2:**
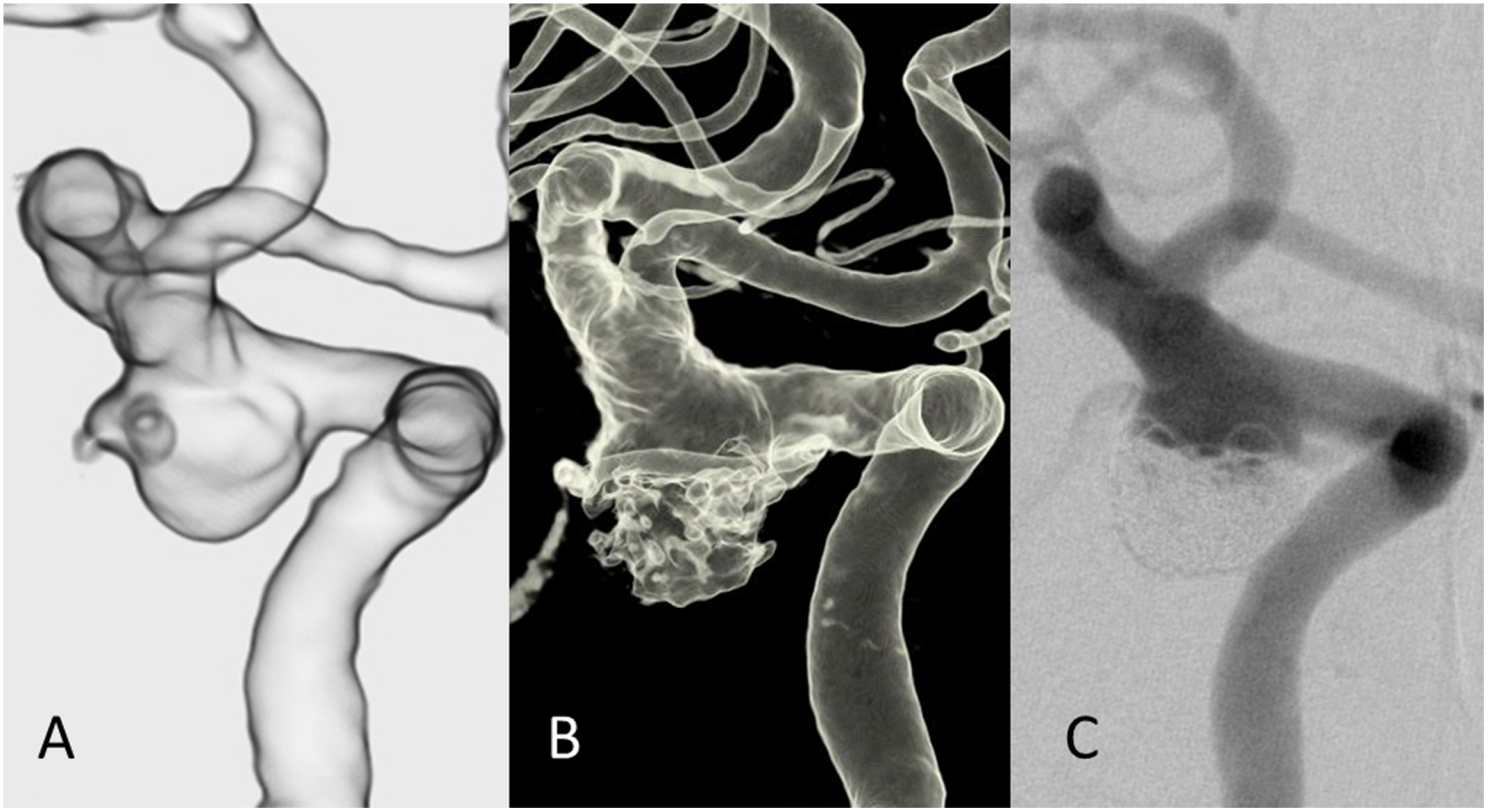
Illustrative case example of a re-canalized left internal carotid artery-posterior communicating artery aneurysm previously treated with stent-assisted coil embolization. **(A)** The VR reconstruction of the TR MRA image after the window level adjustment. **(B)** The 3D reconstruction of the DSA. **(C)** The 2D-DSA image of the same patient. The configuration of the residual aneurysm was better depicted in the TR-MRA image compared to the DSA images. The residual aneurysm in the 3D DSA has an irregular shape, which is presumably caused by metal artifact. Note the size of the residual component in TR-MRA was slightly larger compared to that in 2D DSA image despite the size of parent artery was consistent between the two modalities.

**Fig 3:**
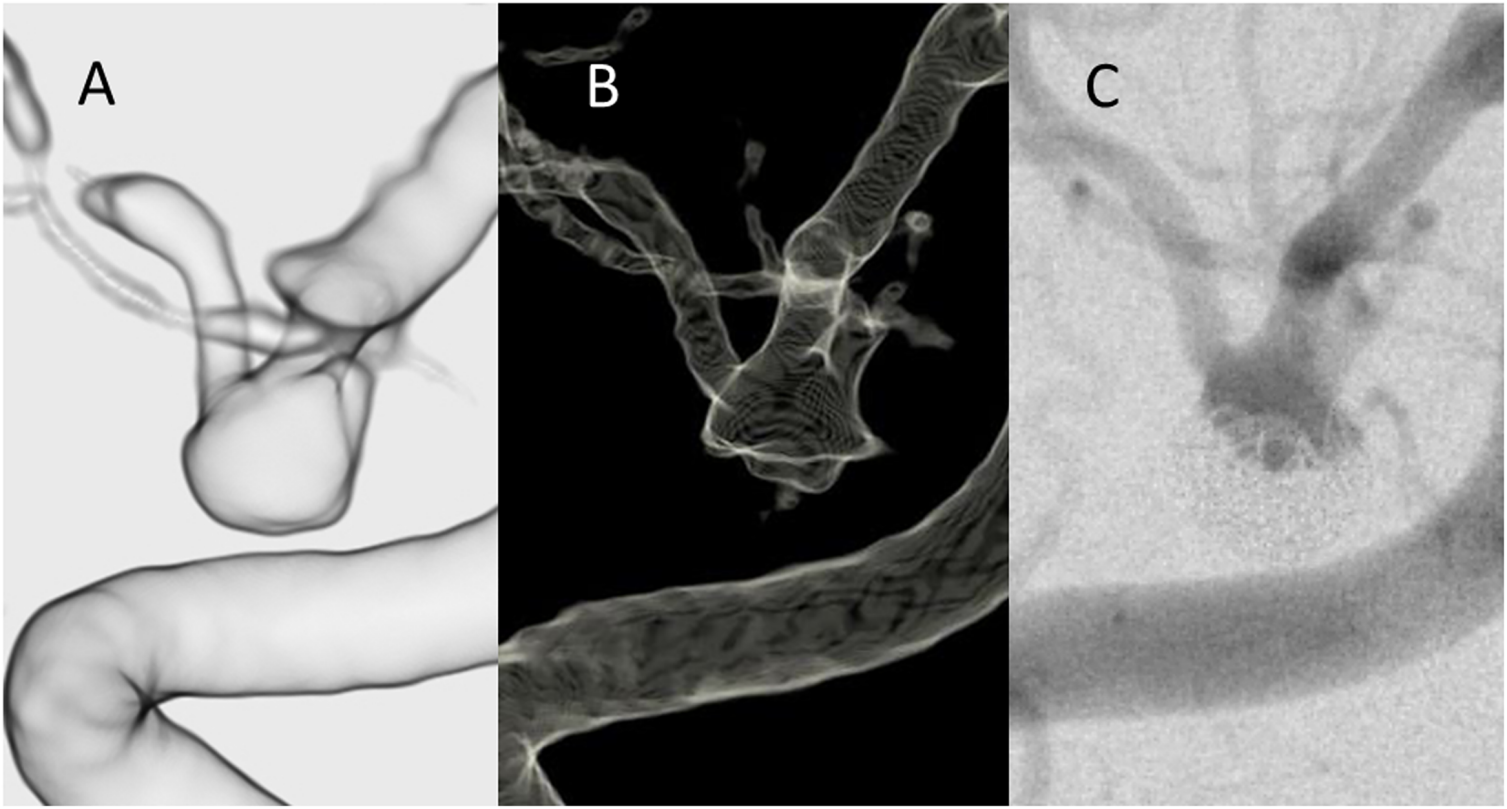
Illustrative case example of a middle cerebral artery aneurysm treated with coil embolization. **(A)** The VR reconstruction of the TR-MRA image after the window level adjustment. **(B)** The 3D reconstruction of the DSA. **(C)** The 2D-DSA image of the same patient. The size of the residual component looks larger in the TR-MRA images than that in the DSA images despite the size of the parent artery was consistent between the two modalities.

The VR images of TR-MRA were successfully created in all 97 aneurysms. The typical VR images of TR-MRA and DSA of post-coiling aneurysms were shown in Figs 2 and 3. Fig 2 shows representative images of a re-canalized left internal carotid artery aneurysm of TR-MRA and DSA. Visualization of the parent artery and the residual aneurysm was well preserved in both modalities. The image findings in both modalities were similar although the 3D DSA showed image degradation in the residual component, presumably due to the metal artifact from the coils. The 2D DSA demonstrated slightly less volume of the residual component compared to the TR-MRA. On the other hand, the size of the parent artery in TR-MRA was consistent with that in DSA.

Fig 3 shows another representative case of a right middle cerebral artery aneurysm demonstrated a residual aneurysm. The TR-MRA showed clear contours of the residual aneurysm. The DSA images in both 2D and 3D demonstrated a relatively smaller residual component. Again, the size of the parent artery in the TR-MRA image was consistent with that in the DSA images.

Overall, the reconstructed VR images provided clear contours of the residual aneurysms and contributed to the understanding of the projection, shape, and size of the residual component.

### Qualitative analysis; Detection sensitivity of the TR-MRA compared to DSA

The results showed that the sensitivity, specificity, positive predictive value, and negative predictive value of TR-MRA were 100% (95%CI: 63–100%), 97% (95%CI: 90–99%), 73% (95%CI: 39–94%), and 100% (95%CI: 96–100%), respectively (Table 1). The unweighted kappa between the two raters was 0.52 (moderate agreement), with an agreement of 87.6%. Furthermore, in the inter-modality agreement between TR-MRA and DSA, the kappa between the two modalities was 0.84 (almost perfect agreement), with an agreement of 96.9%.

**Table 1:** The results of qualitative analysis; Detection sensitivity of the TR-MRA compared to DSA.

	Se (95% CI)	Sp (95% CI)	PPV (95%CI)	NPV (95%CI)
TR-MRA	1.00 (0.63–1.00)	0.97 (0.90–0.99)	0.73 (0.39–0.94)	1.00 (0.96–1.00)

Note: The sensitivity, specificity, positive predictive value, and negative predictive value of TR-MRA for detecting re-canalized aneurysm (DSA as the criterion standard)

Abbreviations: TR-MRA, time-resolved magnetic resonance angiography; DSA, digital subtraction angiogram; Se, Sensitivity; Sp, Specificity; PPV, Positive Predictive Value; NPV, Negative Predictive Value

### Quantitative analysis comparing the size of residual aneurysms between the TR-MRA and DSA

**Fig 4:**
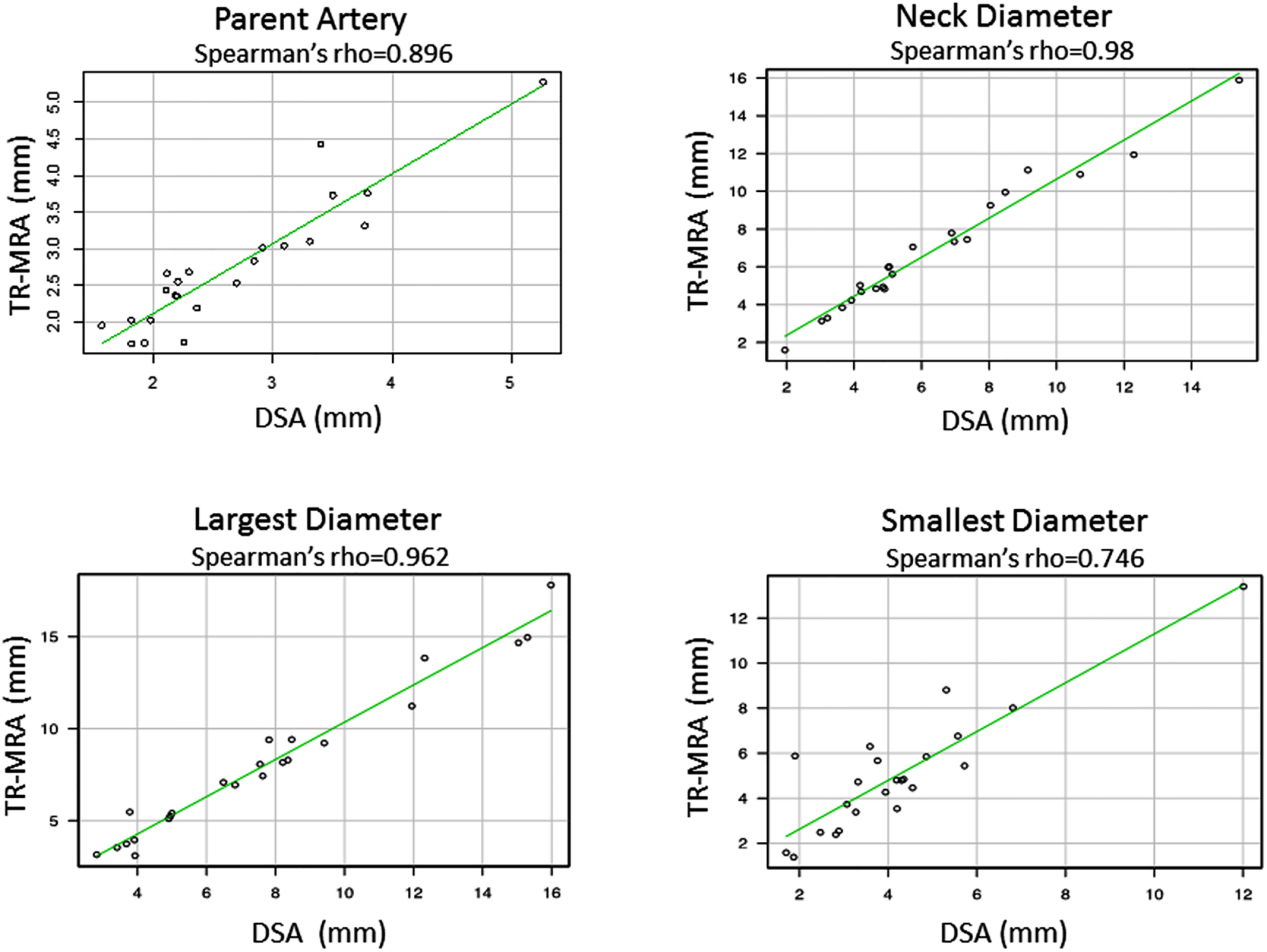
Quantitative analysis comparing the size of residual aneurysm in TR-MRA and DSA. The size measurement of each parameter including the size of parent artery (upper left), the largest diameter (lower left), the neck diameter (upper right), and the smallest diameter (lower right) between the two imaging modalities were strongly correlated (Spearman’s rho = 0.896,0.962,0.98 and 0.746 respectively).

The 23 aneurysms (23.7%) diagnosed with residual neck/aneurysm on follow-up DSA or MRI were selected the quantitative analysis. First, to evaluate the correlation between the two groups, spearman’s rank correlation coefficient was used for statistical analysis. The largest diameters and smallest diameter in the TR-MRA and the DSA were strongly correlated (Spearman’s rho = 0.962 and 0.746 respectively). Similarly, the neck diameters and parent artery were strongly correlated (Spearman’s rho = 0.980 and 0.896 respectively). (Fig 4).

Second, to examine differences in size measurements between the two groups, the statistical analysis was performed using the Wilcoxon signed-rank test. The summary of the quantitative analysis was shown in Table 2. Mean largest diameter of the TR-MRA was 8.05mm (95% Confidence interval (CI), 6.38–9.72mm) for TR-MRA and 7.72 mm (95%CI: 6.09–9.35 mm) for DSA. Similarly mean smallest diameter of the TR-MRA was 4.99 (95% Confidence interval (CI), 3.92–6.06 mm) for TR-MRA and 4.19 mm (95%CI: 3.32–5.06 mm) for DSA. In both parameters, TR-MRA showed statistically larger values compared to those of the DSA (*p* = 0.0004 and *p* = 0.007 respectively). On the other hand, mean value of the parent artery in TR-MRA was 2.75 mm (95%CI, 2.39–3.11mm) for TR-MRA and 2.66mm (95% CI: 2.31–3.01mm) for DSA. Similarly, the neck size in TR-MRA was 6.82 mm (95%CI, 5.43–8.19mm) for TR-MRA and 6.29 mm (95% CI: 4.98–7.6mm) for DSA. Both parameters showed statistically no significance (*p* = 0.260 and *p* = 0.069 respectively)

**Table 2:** The mean size of residual aneurysm comparing TR-MRA and DSA.

	TR-MRA	DSA	Spearman’s rho	p-value*
Parent artery (mm) (95% CI)	2.75 (2.39–3.11)	2.66 (2.31–3.01)	0.896	0.260
Neck (mm) (95% CI)	6.82 (5.43–8.19)	6.29 (4.98–7.6)	0.980	0.069
Largest diameter (mm) (95% CI)	8.05 (6.38–9.72)	7.72 (6.09–9.35)	0.962	0.0004
Smallest diameter(mm) (95% CI)	4.99 (3.92–6.06)	4.19 (3.32–5.06)	0.746	0.007

* = difference between TR-MRA vs DSA (Wilcoxon signed-rank test)

Abbreviations: TR-MRA = time resolved MRA, DSA = digital subtraction angiography

## Discussion

The method of imaging follow-up for aneurysms treated with endovascular technique remains debatable [7]–[8]. Although DSA remains the gold standard of imaging modalities [6], [9], MRI, particularly TOF-MRA, is now widely used for imaging follow-up because it is easy to use and is less invasive [8]. Meanwhile, studies have indicated that TOF-MRA sometimes underestimates the residual aneurysm due to the influence from imaging artifacts e.g. venous flow and susceptibility artifacts from metallic materials [10]. Therefore, other MRI sequences with higher detection sensitivity have been investigated in the past.

Studies have indicated that TR-MRA reduced influence from aforementioned imaging artifacts compared to TOF-MRA [11]. However, little has been reported on the optimal post-processing method to delineate residual aneurysms using the TR-MRA. In this series, a post-processing image was created using an independent 3D workstation to improve the visualization of a residual aneurysm [12]. To minimize inter-observer variability associated with the window level adjustment and to determine the appropriate window level, the FWHM value was used. The VR reconstruction using the FWHM value contributed to the evaluating the contour of the residual component.

Quantitative analysis revealed that the size of the residual component determined by TR-MRA was slightly larger than that by DSA despite the size of parent artery showed consistency between the two modalities. It is potentially because TR-MRA is known to have less susceptibility artifact induced by the metal implant as compared to other MRI sequences [1], whereas 3D-DSA has limited visualization of the cavity space in the coil mass due to a metal artifact. Another possibility is the TR-MRA simply overestimates the vessel size. It is, however, quite unlikely since the size of the neck and parent artery showed consistency between the two modalities.

Nonetheless, these findings are corroborated by the qualitative analysis, which demonstrates that TR-MRA can detect “major recanalization” with 100% sensitivity and 97% specificity. There were some false positive (3%), but no false negatives (0%), indicating that this imaging method may overestimate, but quite unlikely underestimate, the volume of residual aneurysm compared to DSA. Therefore, this imaging modality can be an effective screening tool for detecting re-canalization of a treated aneurysm before considering DSA.

It should be noted that the 3D workstation used in this study, Aquarius iNtuition^®^, is characterized by its unique rendering method called the “Image-Order Rendering”, which is designed to prevent the degradation of the image quality during the 3D reconstruction process and provide clearer images. The reconstructed VR images showed the clear contours of the artery, thus the shape and projection of the residual aneurysm were clearly visualized. This helps evaluators assess the configuration of residual component in each treated aneurysm.

The TR-MRA, however, has its own limitations. For instance, the signal intensity of small / peripheral vessels tends to decrease compared to those of other sequences like TOF. Insufficient signal-to-noise ratio can lead to image degradation. Moreover, inappropriate selection of the time-phase to reconstruct the 3D images can also lead to image degradation. Lastly, the current study was performed using a 1.5 T MRI. Using an MRI with a stronger magnetic field, e.g. 3.0 T, can increase the signal-to-noise ratio and may solve part of the limitations [13]. Further studies using a 3.0T MRI are highly recommended.

A linear contrast agent was used when this study was conducted. However, with the increasing reports about their side effects and the accumulation in the body [14], we believe that macrocyclic agents, instead, should be recommended to reduce the risk of adverse events associated with the contrast agent. It is also important that MRA with contrast use should not be performed routinely, but rather performed on limited occasions e.g. TOF-MRA images are insufficient to delineate the residual component of the treated aneurysms.

## Conclusions

Using the full-width at half-maximum (FWHM) value to optimize the window level adjustment, the reconstructed TR-MRA images provided improved visualization with consistent measurement values of the residual aneurysm. The size of the residual component observed in the TR-MRA was larger compared to that in the DSA whereas the size of neck and the parent artery showed consistency between the two modalities. This image processing technique can be used as an effective screening tool for evaluating residual component in post-coiling aneurysms.

## Supporting information

S1 Table Measurement values of residual aneurysm (raw data) and parent artery. **A**. **Residual aneurysm measured by TR-MRA:** The measurement values of three parameters 1) Neck length, 2) Largest diameter, 3) Smallest diameter as well as the volume of residual aneurysm, which was calculated by the image-processing application “Aquarius iNtuition^®^” are listed. **B**. **Residual aneurysm measured by DSA:** The measurement values of the same parameters described above were listed. **C**. **Diameter of the parent artery:** The size of the parent artery at the proximal neck was compared between the TR-MRA and the 2D DSA.
(PDF)
