# Supplementary material for: Time-resolved magnetic resonance angiography (TR-MRA) for the evaluation of post coiling aneurysms; A quantitative analysis of the residual aneurysm using full-width at half-maximum (FWHM) value
Source: PLoS One. 2018 Sep 7;13(9):e0203615. doi: 10.1371/journal.pone.0203615 (PMC6128576; doi:10.1371/journal.pone.0203615)
Supplement: S1 Table — A. Residual aneurysm measured by TR-MRA: The measurement values of three parameters 1) Neck length, 2) Largest diameter, 3) Smallest diameter as well as the volume of residual aneurysm, which was calculated by the image-processing application “Aquarius iNtuition®” are listed. B. Residual aneurysm measured by DSA: The measurement values of the same parameters described above were listed. C. Diameter of the parent artery: The size of the parent artery at the proximal neck was compared between the TR-MRA and the 2D DSA. (PDF) [file pone.0203615.s001.pdf]

A.Residual aneurysm measured by TR-MRA

| No. | Neck (mm) | Largest diameter (mm) | Smallest diameter (mm) | Volume (mm3) |
|---|---|---|---|---|
| 1 | 3.55 | 1.38 | NA | 0.044 |
| 2 | 4.83 | 5.42 | 4.8 | 0.302 |
| 3 | 15.04 | 21.44 | 11.65 | 2.5419 |
| 4 | 7.796 | 8.076 | 33.823 | 0.399 |
| 5 | 4.94 | 5.26 | 4.84 | 0.1782 |
| 6 | 7.055 | 6.948 | 4.266 | 0.1863 |
| 7 | 7.45 | 11.23 | 6.76 | 0.6337 |
| 8 | 15.89 | 14.96 | 4.46 | 0.9164 |
| 9 | 5.98 | 3.164 | 2.385 | 0.1702 |
| 10 | 10.47 | 8.108 | 7.994 | 0.1959 |
| 11 | 5.03 | 5.48 | 1.58 | 0.07 |
| 12 | 7.429 | 7.439 | 5.876 | 0.7878 |
| 13 | 11.94 | 14.67 | 8.806 | 0.8186 |
| 14 | 9.26 | 13.84 | 8.01 | 0.7707 |
| 15 | 3.844 | 5.122 | 3.533 | 0.2084 |
| 16 | 4.848 | 7.086 | 5.669 | 0.4277 |
| 17 | 6.604 | 7.25 | 6.172 | 0.3726 |
| 18 | 4.689 | 3.754 | 2.546 | 0.1645 |
| 19 | 3.296 | 3.959 | 4.722 | 0.157 |
| 20 | 4.232 | 9.415 | 5.843 | 0.382 |
| 21 | 6.015 | 8.17 | 5.437 | 0.537 |
| 22 | 11.13 | 9.221 | 3.732 | 0.4456 |
| 23 | 3.14 | 3.11 | 2.48 | 0.1133 |

B.Residual aneurysm measured by DSA

| No. | Neck (mm) | Largest diameter (mm) | Smallest diameter (mm) | Volume (mm3) |
|---|---|---|---|---|
| 1 | 1.95 | 3.41 | 1.87 | 0.044 |
| 2 | 4.9 | 5 | 2.2 | 0.2192 |
| 3 | 9.73 | 12.51 | 8.46 | 2.41 |
| 4 | 6.89 | 6.5 | 2.71 | 0.2773 |
| 5 | 4.98 | 3.22 | 2.36 | 0.1844 |
| 6 | 5.7 | 6.8 | 2.2 | 0.1046 |
| 7 | 6.3 | 6.88 | 4.79 | 0.7342 |
| 8 | 3.73 | 10.93 | 5.06 | 0.659 |
| 9 | 2.45 | 3.4 | 2.05 | 0.1178 |
| 10 | 8.47 | 8.7 | 2.95 | 0.2336 |
| 11 | 2.1 | 3.5 | 1.5 | 0.0445 |
| 12 | 6.1 | 8.1 | 1.9 | 0.7493 |
| 13 | 10.1 | 13 | 3.7 | 0.6694 |
| 14 | 6.9 | 9.5 | 4.2 | 0.5784 |
| 15 | 2.3 | 4.36 | 4.19 | 0.2732 |
| 16 | 3.7 | 6.13 | 2.91 | 0.3571 |
| 17 | 4.4 | 8 | 3.8 | 0.3621 |
| 18 | 3.7 | 3.5 | 1.9 | 0.1383 |
| 19 | 2.9 | 4.6 | 3 | 0.0976 |
| 20 | 4.7 | 4.68 | 4.05 | 0.3515 |
| 21 | 4 | 9.9 | 5.4 | 0.5409 |
| 22 | 6.9 | 8.5 | 2.6 | 0.4526 |
| 23 | 3.6 | 4.8 | 2.4 | 0.0615 |

C.Diameter of the parent artery

| No. | TR-MRA (mm) | 3D DSA (mm) |
|---|---|---|
| 1 | 2.53 | 2.69 |
| 2 | 2.83 | 2.84 |
| 3 | 3.01 | 2.91 |
| 4 | 2.02 | 1.97 |
| 5 | 3.1 | 3.31 |
| 6 | 1.7 | 1.81 |
| 7 | 3.76 | 3.79 |
| 8 | 2.36 | 2.18 |
| 9 | 2.43 | 2.1 |
| 10 | 3.31 | 3.77 |
| 11 | 2.02 | 1.81 |
| 12 | 5.28 | 5.27 |
| 13 | 2.19 | 2.36 |
| 14 | 3.04 | 3.09 |
| 15 | 2.68 | 2.29 |
| 16 | 1.72 | 2.25 |
| 17 | 4.43 | 3.4 |
| 18 | 1.95 | 1.56 |
| 19 | 1.71 | 1.92 |
| 20 | 2.55 | 2.2 |
| 21 | 3.73 | 3.5 |
| 22 | 2.66 | 2.11 |
| 23 | 2.35 | 2.19 |
